# Self-Assembled Nanodelivery System with Rapamycin and Curcumin for Combined Photo-Chemotherapy of Breast Cancer

**DOI:** 10.3390/pharmaceutics15030849

**Published:** 2023-03-05

**Authors:** Yanlong Yin, Hong Jiang, Yue Wang, Longyao Zhang, Chunyan Sun, Pan Xie, Kun Zheng, Shaoqing Wang, Qian Yang

**Affiliations:** 1 The Second Affiliated Hospital of Chengdu Medical College (China National Nuclear Corporation 416 Hospital), Center of Scientific Research, Chengdu Medical College, Chengdu 610500, China; 2 School of Bioscience and Technology, Chengdu Medical College, No. 783, Xindu Avenue, Xindu District, Chengdu 610500, China

**Keywords:** nanodelivery system, rapamycin, curcumin, chemo-phototherapy, breast cancer

## Abstract

Nanodelivery systems combining photothermal therapy (PTT) and chemotherapy (CT), have been widely used to improve the efficacy and biosafety of chemotherapeutic agents in cancer. In this work, we constructed a self-assembled nanodelivery system, formed by the assembling of photosensitizer (IR820), rapamycin (RAPA), and curcumin (CUR) into IR820-RAPA/CUR NPs, to realize photothermal therapy and chemotherapy for breast cancer. The IR820-RAPA/CUR NPs displayed a regular sphere, with a narrow particle size distribution, a high drug loading capacity, and good stability and pH response. Compared with free RAPA or free CUR, the nanoparticles showed a superior inhibitory effect on 4T1 cells in vitro. The IR820-RAPA/CUR NP treatment displayed an enhanced inhibitory effect on tumor growth in 4T1 tumor-bearing mice, compared to free drugs in vivo. In addition, PTT could provide mild hyperthermia (46.0 °C) for 4T1 tumor-bearing mice, and basically achieve tumor ablation, which is beneficial to improving the efficacy of chemotherapeutic drugs and avoiding damage to the surrounding normal tissue. The self-assembled nanodelivery system provides a promising strategy for coordinating photothermal therapy and chemotherapy to treat breast cancer.

## 1. Introduction

Breast cancer is one of the most prevalent malignant tumors in the world, with a high mortality rate (15%), ranking second among American female cancer patients [1]. Human epidermal growth factor receptor 2 (HER-2), overexpressed in breast cancer, promotes tumor cell proliferation and invasion by activating the phosphatidylinositol 3-kinase (PI3K)/Akt/mammalian rapamycin target (mTOR) pathway [2]. The mammalian target of rapamycin (mTOR) regulates basic cellular processes, including protein synthesis and cell proliferation, growth, and metabolism, which plays a critical role in the progression of breast cancer [3,4,5,6]. Therefore, mTOR inhibitors have been proposed for treating breast cancer [7,8]. Among them, rapamycin is a natural macrolide drug with antifungal, anti-angiogenesis, and immunosuppressive activities. However, since rapamycin is only able to specifically block the mTOR C 1 complex, its clinical efficacy remains limited, and it is always used in combination with other antineoplastic drugs [9,10].

Curcumin, a natural compound extracted from turmeric, is widely used for its anti-inflammatory, anticancer and anti-infection properties [11,12,13]. Curcumin is a potential target inhibitor of the mTOR pathway, by inhibiting the mTOC 1 and mTOC 2 complexes [14,15,16,17]. In a study investigating the effects of rapamycin and curcumin on epilepsy, curcumin mitigated for the insufficiency of rapamycin with its anti-inflammatory and antioxidation effects [18]. In addition, a clinical study showed that rapamycin and curcumin decreased anti-apoptotic bcl-2 levels, which has important clinical significance in the treatment of B-CLL [19]. Accordingly, curcumin can be used as an adjuvant chemotherapeutic agent for rapamycin.

At present, the main treatment for breast cancer is still chemotherapy. The first-line drug used for targeted therapy against HER-2 is trastuzumab, which has been shown to exert a curative effect through the induction of antibody-dependent cytotoxicity [20]. Although trastuzumab combined with chemotherapy reduces the recurrence rate of HER-2-positive tumors, the cancer, in many patients, has exhibited primary or acquired resistance [21]. At the same time, a clinical trial showed that the combination of the rapamycin derivatives, everolimus and trastuzumab, for early breast cancer patients, not only failed to improve the clinical efficacy of trastuzumab, but triggered corresponding toxic side effects [22]. Given the shortcomings of chemotherapeutic drugs, treatment methods that can enhance the efficacy of chemotherapy need to be further explored, in order to improve patient quality of life [23].

As one of the current clinically representative and non-invasive anticancer therapies, photothermal therapy has shown potential as an adjuvant therapy [24,25,26]. Under external near-infrared light irradiation, the light energy can be converted into local heat by photosensitizers (platinum, gold, iodide, etc.), causing hyperthermia to ablate cancer cells [27,28,29]. Particularly, some small molecule dyes have significant NIR fluorescence imaging properties, and can therefore be used for photothermal therapy with multimodal imaging [30,31]. Among them, the representative indocyanine green (ICG), an FDA-approved near-infrared imaging agent, has been used as a photothermal agent for photothermal therapy [32,33]. As an analog of ICG, IR820 has the advantages of increased near-infrared absorption wavelengths and higher stability than ICG, and is also used as a well-behaved photosensitizer for photothermal therapy [34].

Nanodelivery systems including liposomes, inorganic nanoparticles, polymer nanoparticles, etc., exhibit great advantages in integrating chemotherapy with photothermal therapy, which has become an available technique for breast cancer [35,36,37,38,39]. On the one hand, nanosystems provide a variety of ways for the precise and controlled delivery of therapeutic drugs, by responding to external triggers, such as a thermal response, light response, or pH response [40,41]. On the other hand, nanoparticles (NPs) can be enriched in tumors more effectively, on account of the enhanced permeability and retention (EPR) effect [42]. In previous studies, we utilized nanoparticles IR780/DTX-PCEC@RBC to provide PTT for targeted imaging and ablating of MCF-7 bearing tumors, which enhanced the cytotoxicity of the chemotherapeutic drug docetaxel [43]. The combination of PTT and chemotherapy could enhance the sensitivity of cancer cells to chemotherapeutic drugs, showing great potential in cancer therapy and in overcoming tumor resistance [44,45].

This study utilizes the indocyanine green analog IR820, rapamycin (RAPA), and curcumin (CUR), to build a new nanodelivery system and achieve the purpose of combining photothermal therapy and chemotherapy for breast cancer.

## 2. Materials and Methods

### 2.1. Materials

Rapamycin was purchased from Dalian Meilun Biological Technology Co., Ltd. (Dalian, China). IR820, 3-(4,5-dimethylthiazol-2-yl)-2,5-diphenyltetrazolium bromide (MTT), polyoxide castor oil, and coumarin 6 were supplied from Sigma-Aldrich (Shanghai, China). Dimethyl sulfoxide (DMSO), Tween 80 (medical-grade), and curcumin were obtained from Aladdin Biochemical Technology Co., Ltd. (Shanghai, China). RPMI 1640 and trypsin were purchased from HyClone (Logan, UT, USA). Fetal bovine serum (FBS) was purchased from Life Technologies GmbH (Darmstadt, Germany). Saline was purchased from Sichuan Kelun Pharmaceutical Co., Ltd. (Chengdu, China).

### 2.2. Preparation of the IR820-RAPA/CUR NPs

The nanoparticles were prepared by the self-assembly method. Briefly, a mixture including 6 mg RAPA and 3 mg CUR was dissolved by DMSO. Then the above solution was dropped into 3 mL of IR820 aqueous solution (1 mg/mL) with vigorous stirring (900 rpm/min) for 90 min. Next, the reaction solution was transferred to an activated dialysis bag (WM: 1000 KD), and immersed in 2 L deionized water for dialysis for 4 h. All of the fluid on the outside of the dialysis bag was replaced every 1 h. Finally, the IR820-RAPA/CUR NPs were obtained after centrifugation (10,000 rpm, 10 min) and resuspension with deionized water.

### 2.3. Characterization of the IR820-RAPA/CUR NPs

The particle size, polymer dispersity index (PDI), and potential of the NPs were detected by a dynamic light scattering instrument (Nano-ZS90, Malvern, UK). The particle size stability of the IR820-RAPA/CUR NPs in different temperatures and medias was investigated. The morphology of the nanoparticles was characterized by a transmission electron microscope (TEM) (Tecnai G2 F20 S-TWIN, FEI, Columbia, SC, USA) and a scanning electron microscope (SEM) (Gemini 300, Carl Zeiss AG, Oberkochen, Germany). The crystallinity and chemical bonds of the free RAPA, free IR820, free CUR and IR820-RAPA/CUR NPs were examined by X-ray diffraction (XRD) (DX-2700BH, PANalytical B.V., Almelo, The Netherlands) and Fourier-transform infrared (FTIR) (Nicolet 6700, Thermo Scientific, Waltham, MA, USA), respectively. The Ultraviolet (UV) profiles of the free IR820, free CUR, free RAPA, and the IR820-RAPA/CUR NPs were scanned by a UV spectrophotometer (Specord 50 plus, Analytik Jena AG, Jena, Germany). The absorbance changes of the nanoparticles within a week were examined. The loading efficiency (LE) of the rapamycin was detected by the HPLC method. The conditions of the HPLC detection were as follows, acetonitrile and pure aqueous solution as the liquid phase (66% of acetonitrile, 34% of pure water), a UV detection wavelength of 278 nm, a sample intake volume of 10 μL, a flow rate of 1.0 mL/min, and a column temperature of 40 °C.

### 2.4. In Vitro Release

The in vitro release of the RAPA and CUR was investigated. First, 1 mL of IR820-RAPA/CUR NPs was put into a dialysis bag (WM: 8000–14,000 KD) and immersed in 20 mL of PBS solution (pH = 5.5 or pH = 7.4), containing 0.1% (*v*/*v*) of Tween 80, with thermostatic shaking (100 rpm, 37 °C). The release fluids were taken, to detect the content of RAPA and CUR. The cumulative release amount (C_A_) was calculated according to Equation (1).

(1) $$C_{A}(\%)=\frac{M_{t}}{M_{total}}\times 100\%$$

In this equation, M_t_ stands for the amount of the drug released from the nanoparticles at different times, and M_total_ stands for the total amount of the drug in the nanoparticles.

### 2.5. Photothermal Performance of the IR820-RAPA/CUR NPs

First, volumes of 0.5 mL of PBS, free IR820, and IR820-RAPA/CUR NPs solutions (IR820: 20 μg/mL) were added into 1 mL Eppendorf tubes, and irradiated with a near-infrared laser (808 nm, 1.0 W/cm^2^), for 5 min. The temperature changes and near infrared images were captured every 30 s, using an infrared thermal imaging camera (Tis20, Fluke Corporation, Everett, WA, USA).

In addition, different IR820 concentrations of IR820-RAPA/CUR NPs, and different laser powers, were used, according to the same method, to investigate the temperature changes.

### 2.6. In Vitro Studies

Murine-derived breast cancer cells 4T1 were a gift from Prof. Jinrong Peng at the State Key Laboratory of Biotherapy and Cancer Center, West China Hospital, Sichuan University (Chengdu, China). The cells were cultured in RPMI 1640, containing 10% FBS and 1% penicillin and streptomycin, with a CO_2_ incubator (37 °C, 5% CO_2_).

#### 2.6.1. Cytotoxicity Assay

The 4T1 cells were implanted into 96-well plates (5 × 10^3^ cells/well). After the cells were fully adhered, 100 μL of free IR820, free RAPA, free CUR, RAPA + CUR, and IR820-RAPA/CUR NPs, diluted with culture medium to various concentrations, were added to each well. An MTT assay was executed after incubation for 24 h. Then, a microplate reader (VICTOR Nivo™, Perkinelmer, Inc., Waltham, MA, USA) was applied, to detect the optical density (OD) values. The synergy indexes (CI) of the RAPA and CUR were calculated using the CompuSyn software 2.0.

#### 2.6.2. Cellular Internalization

In order to compare the differences between the small molecule drugs, the small molecule self-assembly systems, and the polymer micelles entering the 4T1 cells, we fluorescently labeled the IR820-RAPA/CUR NPs and polymer micelles with coumarin 6 (C6). The preparation of the micelles referenced the research of Zhang et al. [46]. Then, the 4T1 cells, seeded in 6-well plates, were treated with coumarin-labeled IR820-RAPA/CUR NPs (NPs-C6), coumarin-labeled micelles (micelles-C6), and free coumarin 6 for 0.5 h, 1 h, and 4 h, respectively. The cells were washed and then observed by microscopy (Olympus IX 83, Olympus Optical Company Ltd., Tokyo, Japan).

#### 2.6.3. Cell Apoptosis

The 4T1 cells seeded in 6-well plates were treated with DMEM, free RAPA, free CUR, RAPA + CUR, and IR820-RAPA/CUR NPs. The IR820-RAPA/CUR NPs + laser group was irradiated with near-infrared light (808 nm, 1 W/cm^2^, 5 min) at 20 h, and then returned to the incubator for another 4 h. The cells in the well plates were digested with trypsin without ethylenediaminetetraacetic acid disodium salt (EDTA-2Na). The collected cells were washed, and treated by an apoptosis kit. Finally, the apoptosis of the cells was detected by flow cytometry (NovoCyte 3130, ACEA Biosciences Inc., San Diego, CA, USA).

### 2.7. In Vivo Studies

#### 2.7.1. In Vivo Animal Tumor Models

Balb/c female mice (18–20 g) were provided by Chengdu Dashuo Experimental Animal Co., Ltd. (Chengdu, China). All animals were handled in accordance with the protocol procedures approved by the Ethics Committee of Chengdu Medical College (2021–2022). Each mouse was subcutaneously injected with 2 × 10^6^ 4T1 cells, in the right flank, for modeling.

#### 2.7.2. In Vivo Photothermal Performance of IR820-RAPA/CUR NPs

The 4T1 tumor-bearing mice were injected intravenously with saline and IR820-RAPA/CUR NPs (IR820: 1.5 mg/kg), respectively. After 24 h administration, the mice were anesthetized by intraperitoneal injection of 200 μL of 5% (*w*/*v*) chloral hydrate. All mice were irradiated by an 808 nm near-infrared light (1.5 W/cm^2^) for 5 min. The near infrared images and temperature changes were recorded by an infrared imaging device.

#### 2.7.3. Antitumor Efficacy and Histopathological Analysis

The mice were divided into four groups (*n* = 3), to evaluate the chemotherapy and photothermal therapy: (1) PBS; (2) free RAPA + free CUR (RAPA: 9.0 mg/kg and CUR 6.0 mg/kg); (3) IR820-RAPA/CUR NPs (RAPA: 9.0 mg/kg, and CUR 6.0 mg/kg); and (4) IR820-RAPA/CUR NPs with laser (RAPA: 9.0 mg/kg, and CUR 6.0 mg/kg). All experimental groups were administered therapy on days 0, 2, 4, and 7, and PTT was performed the day after the first administration. The body weight and tumor size of all mice were measured every two days.

On the twelfth day after drug administration, mice were euthanized, and their tumor and major organs (hearts, livers, spleens, lungs, and kidneys) were removed for histopathological analysis.

#### 2.7.4. In Vivo Drug Dose Investigation

To evaluate the effect of the nanoparticle concentration on the chemotherapy and photothermal therapy, the mice were divided into 7 groups: (1) saline; (2) IR820-RAPA/CUR NPs (RAPA: 4.5 mg/kg and CUR 3.0 mg/kg); (3) IR820-RAPA/CUR NPs (RAPA: 4.5 mg/kg and CUR 3.0 mg/kg) with laser; (4) IR820-RAPA/CUR NPs (RAPA: 9 mg/kg and CUR: 6 mg/kg); (5) IR820-RAPA/CUR NPs (RAPA: 9 mg/kg and CUR: 6 mg/kg) with laser; (6) IR820-RAPA/CUR NPs (RAPA: 18 mg/kg and CUR: 12 mg/kg); and (7) IR820-RAPA/CUR NPs (RAPA: 18 mg/kg and CUR: 12 mg/kg) with laser. All experimental groups were administered therapy on days 0, 2, 4, and 7, and PTT was performed in the (3), (5), and (7) groups the day after the first administration. The body weight and tumor size of 4T1 tumor-bearing mice were recorded.

## 3. Results

### 3.1. Characterization of the IR 820-RAPA/CUR NPs

**Figure 1:**
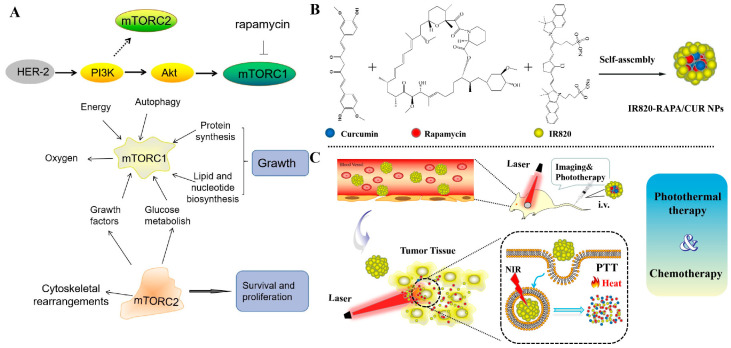
(**A**) Schematic diagram of mTOR pathway and functions of mTOR C1 and mTOR C2. Schematic diagram of IR820-RAPA/CUR NPs constitution (**B**), and in vivo antitumor process (**C**).

**Figure 2:**
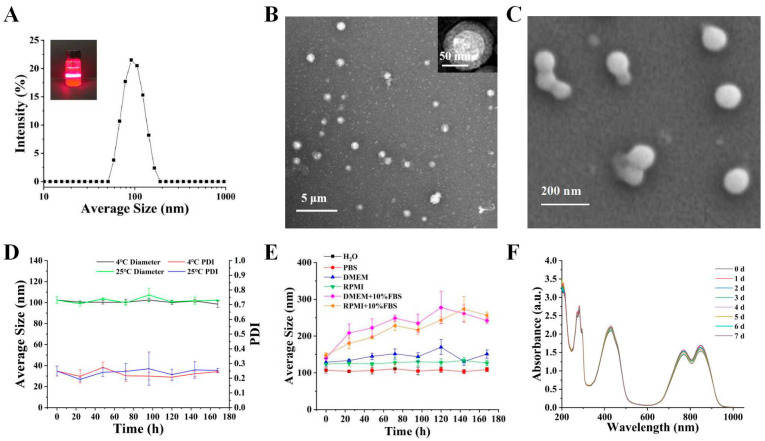
Characterization of IR820-RAPA/CUR NPs. (**A**) Tyndall effect and particle size of IR820-RAPA/CUR NPs. (**B**) Transmission electron microscope image (TEM) and (**C**) scanning electron microscope image (SEM) of IR820-RAPA/CUR NPs. (**D**) Particle size and PDI of IR820-RAPA/CUR NPs at 4 °C and 25 °C. (**E**) Particle size changes of IR820-RAPA/CUR NPs in H_2_O, PBS, DMEM, RPMI 1640, DMEM + 10% FBS, and RPMI 1640 + 10% FBS. (**F**) Changes in absorbance of IR820-RAPA/CUR NPs after a week in the dark.

**Figure 3:**
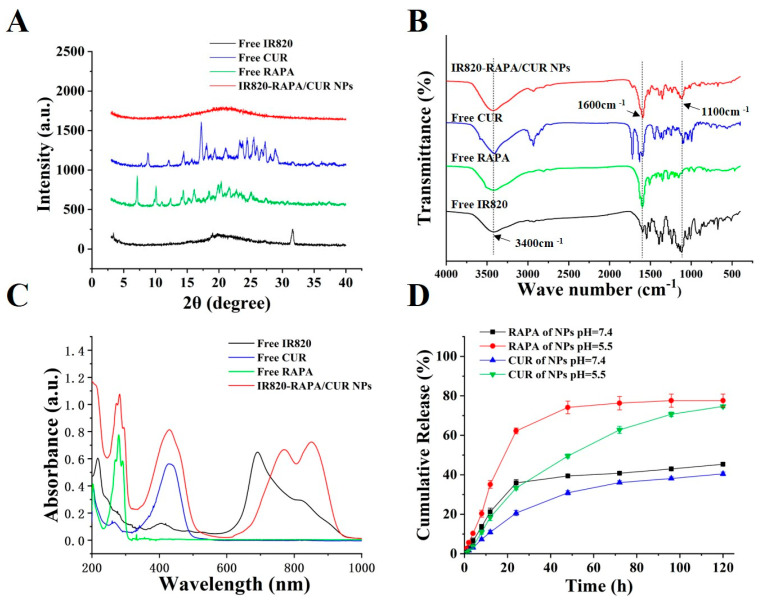
(**A**) XRD spectra, (**B**) FTIR spectra, and (**C**) UV-Vis-NIR absorption spectra of free CUR, free RAPA, free IR820, and IR820-RAPA/CUR NPs. (**D**) In vitro release curve of IR820-RAPA/CUR NPs, at pH 5.5 and pH 7.4.

In this study, we prepared a novel nanodrug, IR820-RAPA/CUR NPs, via a self-assembly approach (Figure 1). The IR820-RAPA/CUR NPs possessed a uniform size, of 109.6 ± 1.73 nm, and that of the PDI was 0.25 ± 0.024 (Figure 2A). The surface charge of the IR820-RAPA/CUR NPs was −20.03 ± 0.48 mV. The LE (%) of the RAPA, CUR, and IR820 in the IR820-RAPA/CUR NPs were 34.94 ± 0.048%, 26.69 ± 0.53%, and 38.38 ± 0.50%, respectively. The transmission electron microscope (Figure 2B) and scanning electron microscope (Figure 2C) results showed that the IR820-RAPA/CUR NPs presented a smooth spherical surface. The UV spectrum of the IR820-RAPA/CUR NPs showed typical absorption peaks at 278 nm, and 426 nm for RAPA, and CUR, respectively (Figure 3C). The free IR820 had a maximum absorption at ~692 nm. However, the IR820-RAPA/CUR NPs absorption spectrum underwent a significant redshift, with absorption peaks appearing at 770 nm and 851 nm. Besides, there was no new chemical bond formation in the IR820-RAPA/CUR NPs, as proved by FTIR (Figure 3B).

The IR820-RAPA/CUR NPs were stored for a week at 4 °C and 25 °C, with no significant change in the particle size and PDI (Figure 2D), and with admirable size stability in water, PBS, DMEM, and RPMI. However, the particle size of the IR820-RAPA/CUR NPs in the DMEM + 10% FBS and RPMI 1640 + 10% FBS tended to increase, which might be due to the electrostatic adsorption occurring between the nanoparticles and the proteins in the serum, resulting in the aggregation of nanoparticles. There was no significant change in the absorbance of the IR820-RAPA/CUR NPs during storage, indicating good photostability (Figure 2F).

As shown in Figure 3A, there were typical peaks of free CUR, free RAPA, and free IR820 in the XRD spectrum. However, all peaks of the three drugs completely disappeared in the IR820-RAPA/CUR NPs, implying that the nanoparticles were in an amorphous state, due to interactions between the components [47,48].

### 3.2. Drug Release Behavior

The in vitro release behavior of the IR820-RAPA/CUR NPs, at pH =5.5 and pH = 7.4, was examined. As shown in Figure 3D, the IR820-RAPA/CUR NPs exhibited a pH-responsive release property. At pH = 7.4, there was a 45.32 ± 0.84% rapamycin, and 40.50 ± 0.94% curcumin release, from IR820-RAPA/CUR NPs at 120 h. However, under the weak acid conditions of pH = 5.5, the RAPA and CUR showed a more rapid release. At the same incubation time, there was a 77.59 ± 3% rapamycin, and 74.66 ± 0.80% curcumin release, from the IR820-RAPA/CUR NPs. The above results indicate that the release of rapamycin and curcumin increased as the pH decreased, which is beneficial to the release of drugs in weakly acidic tumor sites [49].

### 3.3. In Vitro Photothermal Properties

**Figure 4:**
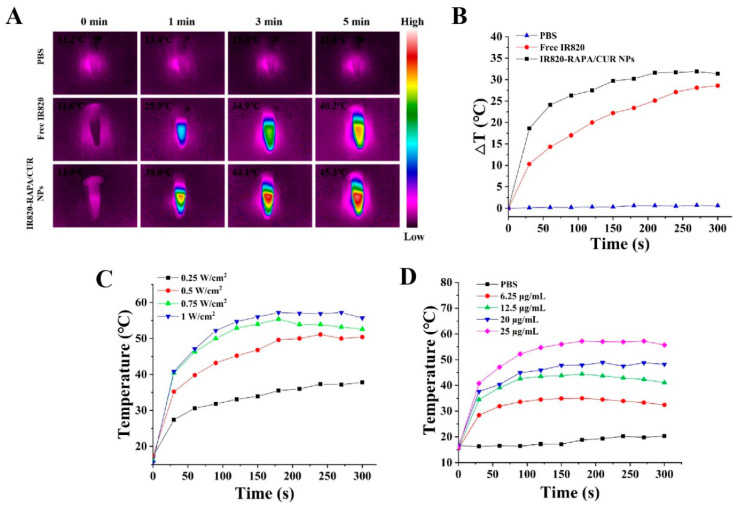
(**A**) Infrared thermal imaging and (**B**) heating curve of PBS, free IR820, and IR820-RAPA/CUR NPs solutions irradiated at 808 nm, 1.0 W/cm^2^, for 5 min. (**C**,**D**) Photothermal heating curves of the IR820-RAPA/CUR NPs solution with different concentrations and various power intensities.

To assess the photothermal properties of the free IR820 and IR820-RAPA/CUR NPs, NIR was implemented in PBS, free IR820, and IR820-RAPA/CUR NPs. The temperature of the IR820-RAPA/CUR NPs rose to 45.3 °C, higher than the 40.2 °C of free IR820, and no significant temperature change was observed in the PBS (Figure 4A,B), indicating the excellent in vitro photothermal properties of the IR820-RAPA/CUR NPs. In addition, the temperature of the nanoparticles rose with the increase in the IR820 concentration and laser power, indicating that the IR820-RAPA/CUR NPs had favorable in vitro photothermal performance, and could be used as a photothermal agent for photothermal therapy.

### 3.4. Cytotoxicity Assay

**Figure 5:**
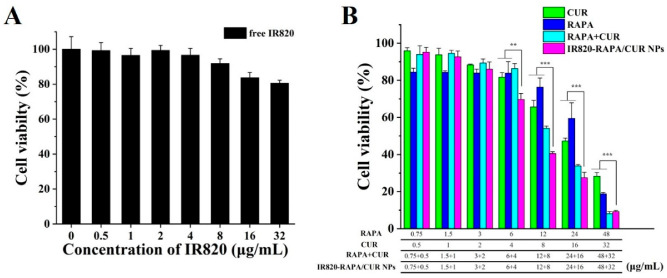
Cytotoxicity of free IR820 (**A**), free RAPA, free CUR, RAPA + CUR, and IR820-RAPA/CUR NPs (**B**) at various concentrations incubated with 4T1 cells for 24 h. (*n* = 6; ** *p* < 0.01, *** *p* < 0.001).

The growth inhibition of the 4T1 breast cancer cells treated by the free RAPA, free CUR, RAPA + CUR, and IR820-RAPA/CUR NPs was investigated by MTT assay (Figure 5A). By controlling the administration ratio of RAPA and CUR to 3:2, the free RAPA, free CUR, RAPA + CUR, and IR820-RAPA/CUR NPs all exhibited concentration-related proliferation inhibition on the 4T1 breast cancer cells. As shown in Figure 5B, when the concentrations of RAPA and CUR were 12 μg/mL and 8 μg/mL, respectively, the 4T1 cell survival rates of the free CUR group, free RAPA group, RAPA + CUR group, and the nanoparticle group were 65.63%, 76.32%, 54.11%, and 40.52%, respectively. The synergy index of the RAPA and CUR at this concentration was 1.23, indicating that the RAPA and CUR were antagonistic at this concentration. Moreover, when the drug concentration was lower than this, the synergy index of each group was greater than 1. However, with the increase in concentration, when the concentrations of RAPA and CUR were 24 μg/mL and 16 μg/mL, respectively, the 4T1 cells survival rates of each group were 47.26%, 59.48%, 33.85%, and 27.47%, respectively. The synergy index of the RAPA and CUR at this concentration was 0.88, and the RAPA and CUR showed a weak synergy effect. When the concentrations of RAPA and CUR were 48 μg/mL and 32 μg/mL, respectively, the cell survival rates of each group were 28.30%, 18.76%, 8.11%, and 9.19%, respectively. The synergy index of the RAPA and CUR at this concentration was 0.23, reflecting a high degree of synergy between the RAPA and CUR.

### 3.5. Cellular Uptake

**Figure 6:**
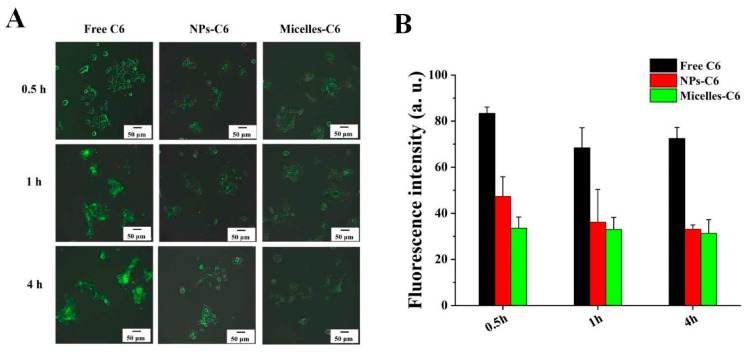
Images (**A**) and mean fluorescence intensity (**B**) of 4T1 cells treated with free C6, NPs-C6, and micelles-C6 at 0.5 h, 1 h, and 4 h.

As shown in Figure 6, at 0.5 h, the green fluorescence in the 4T1 cells of the three groups was weak, indicating that the 4T1 cells had a lower uptake of the free C6, nanoparticles, and micelles at this time. There was a strong green fluorescence of C6 emerging in the free C6 group at 1 h, while only a weaker green fluorescence was observed in the NPs-C6 group and the micelles-C6 group at 1 h and 4 h, indicating that the uptake of the free C6 by 4T1 cells was more rapid than that of the nanoparticles and micelles.

### 3.6. Apoptosis of 4T1 Cells

**Figure 7:**
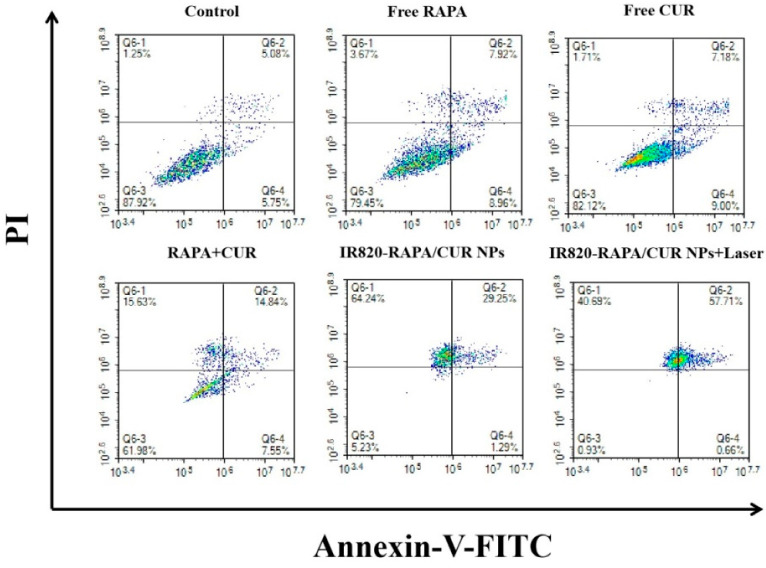
Apoptosis of 4T1 cells detected by flow cytometry of the control group, free RAPA group, free CUR group, RAPA + CUR group, IR820-RAPA/CUR NPs group, and IR820-RAPA/CUR NPs + laser group (*n* = 3).

In this study, the apoptosis of the 4T1 cells induced by the free RAPA, free CUR, RAPA + CUR, IR820-RAPA/CUR NPs, and the IR820-RAPA/CUR NPs + laser was examined. As shown in Figure 7, the apoptosis rates in the control group, free RAPA group, free CUR group, and RAPA + CUR group were 10.83%, 16.88%, 16.18%, and 22.39%, respectively, indicating that RAPA or CUR alone induced the apoptosis of 4T1 cells to a certain extent. The induction of the apoptosis of the 4T1 cells was higher when RAPA was combined with CUR. The apoptotic rates of the cells in the IR820-RAPA/CUR NPs group and the IR820-RAPA/CUR NPs + laser group were 30.54% and 58.37%, respectively, indicating that the nanoparticles significantly increased the apoptosis rate of the 4T1 cells, while the near-infrared laser irradiation better induced apoptosis, which indicates that photothermal therapy can enhance the inhibitory effect of the nanoparticles on the 4T1 cells.

### 3.7. In Vivo Photothermal Imaging

**Figure 8:**
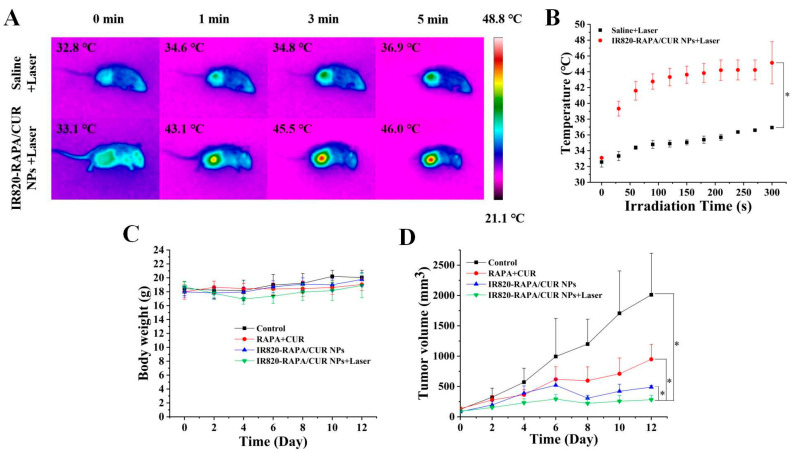
(**A**) Infrared thermal images and (**B**) in vivo photothermal heating curve of 4T1 tumor-bearing mice irradiated by 808 nm laser (1.5 W/cm^3^) for 5 min, after i.v. injection of saline and IR820-RAPA/CUR NPs. (**C**) Body weight changes, (**D**) tumor volume changes of 4T1 tumor-bearing mice within 12 days after i.v. injection of saline, RAPA + CUR, IR820-RAPA/CUR NPs, and IR820-RAPA/CUR NPs + laser. (*n* = 3; * *p* < 0.05).

The photothermal efficacy of the IR820-RAPA/CUR NPs in vivo was investigated. In the IR820-RAPA/CUR NPs + laser group, the tumor temperature of the 4T1 tumor-bearing mice increased from 33.1 °C to 46.0 °C, after near-infrared laser irradiation for 5 min, and the photothermal performance was significantly better than that of saline (Figure 8A,B). At this temperature, the nanoparticles provided mild photothermal therapy.

### 3.8. In Vivo Antitumor Activity Study of the IR820-RAPA/CUR NPs

The in vivo antitumor ability of the chemotherapy and PTT was evaluated. As shown in Figure 8D, the tumors of the tumor-bearing mice in the saline group grew rapidly, while the IR820-RAPA/CUR NPs with or without laser irradiation had a more obvious inhibitory effect on tumor growth within 12 days after administration. Furthermore, the tumors grew more slowly when the 4T1 tumor-bearing mice were treated by IR820-RAPA/CUR NPs with 808 nm laser irradiation, showing superior tumor suppression. The results showed that the introduction of PTT enhanced the antitumor effect of chemotherapy.

### 3.9. In Vivo Study of the Safety

**Figure 9:**
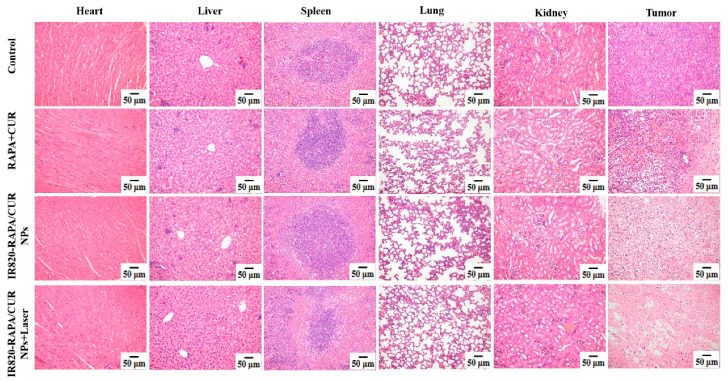
The images of H&E staining in different organs (hearts, livers, spleens, lungs, and kidneys) and tumors of 4T1 bearing mice in each formulation group, after injection of different formulations (scale bar: 50 μm).

As shown in Figure 8C, no significant weight loss occurred in any of the mice within 12 days after administration. The H&E staining results in each group are shown in Figure 9. No significant cell damage or morphological changes were found, indicating that photothermal therapy did not cause significant side effects, while effectively improving the anticancer efficacy of the chemotherapeutic drugs.

### 3.10. Effect of the Nanoparticles’ Dosage on Tumors

**Figure 10:**
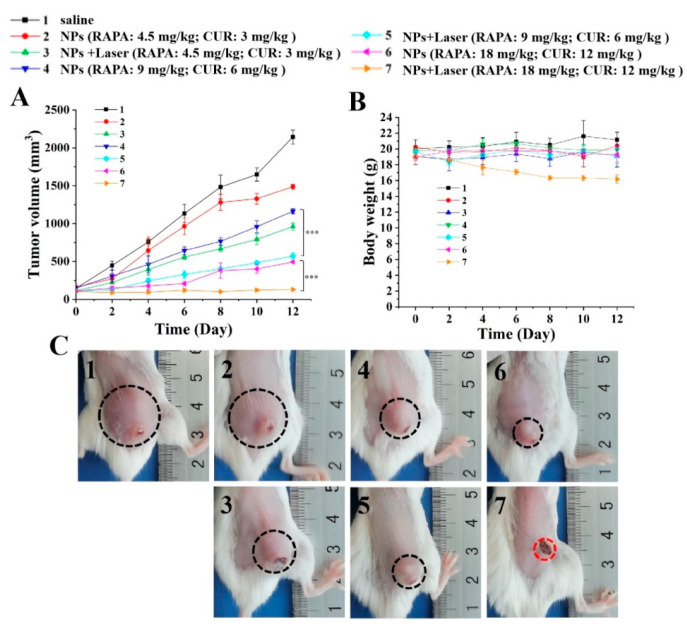
(**A**) Tumor growth curve, (**B**) body weight changes of tumor-bearing mice in the control, low, medium, and high dose groups, within 12 days after administration, and (**C**) tumor photos of each group on the 12th day after administration (*n* = 3; *** *p* < 0.001).

To further investigate the therapeutic effect of different doses of IR820-RAPA/CUR NPs on tumors, the treatment of 4T1 tumor-bearing mice with low, medium, and high doses of nanoparticles, with or without NIR irradiation, was investigated. As shown in Figure 10, the in vivo tumor inhibitory effect of the IR820-RAPA/CUR NPs increased with the increase in the nanoparticle dose, and the tumor sites of the mice treated with the near-infrared laser showed obvious ablation. When the doses of RAPA and CUR were 18 mg/kg and 12 mg/kg, respectively, the addition of NIR enhanced the antitumor effects of the rapamycin and curcumin, and achieved tumor ablation (Figure 10A,C). However, the tumor-bearing mice at this concentration experienced continuous weight loss after the laser irradiation, compared with the group without the laser, indicating that the temperature increase associated with high concentrations had adverse effects on the safety of the mice (Figure 10B).

## 4. Discussion

Photothermal therapy is often combined with other treatment patterns, and is widely used in the treatment of various cancers [50,51,52]. In this study, IR80-RAPA/CUR NPs were prepared via self-assembly, using the photothermal agent IR820, with the chemical medicines, rapamycin and curcumin. During our preparation of the nanoparticles, rapid agitation enhanced the physical interaction between the IR820, RAPA, and CUR, forming ~110 nm spherical nanoparticles (Figure 2A–C). The curcumin was not only a potential chemotherapeutic agent, but also an excellent physical crosslinker that could connect the hydrophobic segment with IR820 and rapamycin, due to its particular symmetrical structure. Two hydrophobic phenyl domains of curcumin co-stacked with that of IR820, to form nanoparticles [53], as confirmed by XRD (Figure 3A). Furthermore, hydrophobic forces and hydrogen bonding might be the factors determining the nanoparticles’ formation (Figure 3B) [54,55].

The UV–Vis–NIR spectrum of the free IR820 had a maximum absorption at ~692 nm (Figure 3C). However, the IR820-RAPA/CUR NPs’ absorption spectrum underwent a significant redshift, with absorption peaks appearing at 770 nm and 851 nm. It was shown that the three small molecules, IR820, CUR, and RAPA, formed nanoparticles by π–π stacking and hydrophobic interactions, causing molecular conformation changes via co-assembly [56,57]. Studies have reported that the strong non-covalent interaction between the aromatic ring in ICG and other hydrophobic drugs caused the red shift of the UV characteristic absorption peak of ICG [58,59]. Therefore, the response of the IR820-RAPA/CUR NPs to the near-infrared laser was more sensitive, resulting in a significant temperature increase compared to the free IR820 under laser irradiation.

Compared with the nanoparticles and micelles, free C6 has a higher lipid solubility and smaller molecular weight, so it can penetrate the cell membrane and enter into cells more quickly. In addition, the hydrophilic groups outside the micelles not only maintain the hydrophilicity of micelles, but also limit the fusion of micelles with the cell membranes.

In this work, the tumor suppression of IR820-RAPA/CUR NPs on 4T1 cells was proved. In addition, we confirmed that PTT could induce mild hyperthermia (46.0 °C) in the 4T1 tumor-bearing mice, after the tail vein injection of IR820-RAPA/CUR NPs with NIR irradiation (Figure 8A,B). The nanoparticles inhibited tumor growth in a dose-dependent manner, which could be due to the synthetic effect of the hyperthermia-based cell necrosis and chemotherapy. In the central region, the tumor tissue underwent thermal ablation, while the incomplete apoptosis in the peripheral region could be cured by the cytotoxic effects of the RAPA and CUR. According to previous reports, mild hyperthermia, at 41–48 °C, could increase the blood flow and permeability of tumor tissues, which is conducive to the enrichment of nanoparticles. In addition, the PTT increased the level of DNA alkylation in cancer cells, and induced apoptosis by increasing the sensitivity of the tumor cells to chemotherapy [60]. In these studies, the biodistribution of the IR820-RAPA/CUR NPs in mice should also be evaluated, to explore the targeting of the nanoparticles. Furthermore, the mechanism of the combined action of rapamycin and curcumin needs to be further explored, by performing immunohistochemistry or real-time-PCR.

## 5. Conclusions

In this study, a self-assembled nanoparticle delivery system, that achieved co-delivery of RAPA and CUR to treat breast cancer, combining photothermal therapy and chemical therapy, was successfully constructed. The particle size, PDI, and LE of the nanoparticles were relatively ideal, with admirable stability, pH responsive ability, and good photothermal performance. The IR820-RAPA/CUR NPs exhibited excellent tumor suppression and suitable safety in vivo. In addition, photothermal therapy enhanced the antitumor effect of chemotherapeutic agents, and achieved tumor ablation at a high dose. In short, IR820-RAPA/CUR NPs might be a promising candidate for breast cancer, via photo-chemotherapy.

## Data Availability

Data is unavailable due to no new data was created.

## References

[1] Siegel R.L., Miller K.D., Fuchs H.E., Jemal A. (2022). Cancer statistics, 2022. CA Cancer J. Clin..

[2] Slamon D.J., Clark G.M., Wong S.G., Levin W.J., Ullrich A., McGuire W.L. (1987). Human breast cancer: Correlation of relapse and survival with amplification of the HER-2/neu oncogene. Science.

[3] Murugan A.K. (2019). mTOR: Role in cancer, metastasis and drug resistance. Semin. Cancer Biol..

[4] Liu G.Y., Sabatini D.M. (2020). mTOR at the nexus of nutrition, growth, ageing and disease. Nat. Rev. Mol. Cell Biol..

[5] Cui J., Shen H.M., Lim L.H.K. (2020). The role of autophagy in liver cancer: Crosstalk in signaling pathways and potential therapeutic targets. Pharmaceuticals.

[6] Vernieri C., Milano M., Brambilla M., Mennitto A., Maggi C., Cona M.S., Prisciandaro M., Fabbroni C., Celio L., Mariani G. (2019). Resistance mechanisms to anti-HER2 therapies in HER2-positive breast cancer: Current knowledge, new research directions and therapeutic perspectives. Crit. Rev. Oncol. Hematol..

[7] Lu C.H., Wyszomierski S.L., Tseng L.M., Sun M.H., Lan K.H., Neal C.L., Mills G.B., Hortobagyi G.N., Esteva F.J., Yu D. (2007). Preclinical testing of clinically applicable strategies for overcoming trastuzumab resistance caused by PTEN deficiency. Clin. Cancer Res..

[8] Yang Q., Xiao Y., Liu Q., Xu X., Peng J. (2020). Carrier-Free Small-Molecule Drug Nanoassembly Elicits Chemoimmunotherapy via Co-inhibition of PD-L1/mTOR. ACS Appl. Biol. Mater..

[9] Guo S., Lin C.M., Xu Z., Miao L., Wang Y., Huang L. (2014). Co-delivery of cisplatin and rapamycin for enhanced anticancer therapy through synergistic effects and microenvironment modulation. ACS Nano.

[10] Dai W., Yang F., Ma L., Fan Y., He B., He Q., Wang X., Zhang H., Zhang Q. (2014). Combined mTOR inhibitor rapamycin and doxorubicin-loaded cyclic octapeptide modified liposomes for targeting integrin alpha3 in triple-negative breast cancer. Biomaterials.

[11] Adamczak A., Ozarowski M., Karpinski T.M. (2020). Curcumin, a natural antimicrobial agent with strain-specific activity. Pharmaceuticals.

[12] Li L., Zhang X., Pi C., Yang H., Zheng X., Zhao L., Wei Y. (2020). Review of curcumin physicochemical targeting delivery system. Int. J. Nanomed..

[13] Chahar M.K., Sharma N., Dobhal M.P., Joshi Y.C. (2011). Flavonoids: A versatile source of anticancer drugs. Pharmacogn. Rev..

[14] Borges G.A., Elias S.T., Amorim B., de Lima C.L., Coletta R.D., Castilho R.M., Squarize C.H., Guerra E.N.S. (2020). Curcumin downregulates the PI3K–AKT–mTOR pathway and inhibits growth and progression in head and neck cancer cells. Phytother. Res..

[15] Wang J., Zhang J., Zhang C.J., Wong Y.K., Lim T.K., Hua Z.C., Liu B., Tannenbaum S.R., Shen H.M., Lin Q. (2016). In Situ proteomic profiling of curcumin targets in HCT116 colon cancer cell line. Sci. Rep..

[16] Beevers C.S., Chen L., Liu L., Luo Y., Webster N.J., Huang S. (2009). Curcumin disrupts the mammalian target of rapamycin-raptor complex. Cancer Res..

[17] Beevers C.S., Li F., Liu L., Huang S. (2006). Curcumin inhibits the mammalian target of rapamycin-mediated signaling pathways in cancer cells. Int. J. Cancer.

[18] Drion C.M., Van Scheppingen J., Arena A., Geijtenbeek K.W., Kooijman L., Van Vliet E.A., Aronica E., Gorter J.A. (2018). Effects of rapamycin and curcumin on inflammation and oxidative stress in vitro and in vivo—In search of potential anti-epileptogenic strategies for temporal lobe epilepsy. J. Neuroinflamm..

[19] Hayun R., Okun E., Berrebi A., Shvidel L., Bassous L., Sredni B., Nir U. (2009). Rapamycin and curcumin induce apoptosis in primary resting B chronic lymphocytic leukemia cells. Leuk. Lymphoma.

[20] Vogel C.L., Cobleigh M.A., Tripathy D., Gutheil J.C., Harris L.N., Fehrenbacher L., Slamon D.J., Murphy M., Novotny W.F., Burchmore M. (2002). Efficacy and safety of trastuzumab as a single agent in first-line treatment of HER2-overexpressing metastatic breast cancer. J. Clin. Oncol..

[21] Rimawi M.F., De Angelis C., Schiff R. (2015). Resistance to anti-HER2 therapies in breast cancer. Am. Soc. Clin. Oncol. Educ. Book.

[22] Campone M., Bachelot T., Treilleux I., Pistilli B., Salleron J., Seegers V., Arnedos M., Loussouarn D., Wang Q., Vanlemmens L. (2021). A phase II randomised study of preoperative trastuzumab alone or combined with everolimus in patients with early HER2-positive breast cancer and predictive biomarkers (RADHER trial). Eur. J. Cancer.

[23] Noe M.H., Wan M.T., Shin D.B., Armstrong A.W., Duffin K.C., Chiesa Fuxench Z.C., Kalb R.E., Menter A., Simpson E.L., Takeshita J. (2019). Patient-reported outcomes of adalimumab, phototherapy, and placebo in the vascular inflammation in psoriasis trial: A randomized controlled study. J. Am. Acad. Dermatol..

[24] Yang Q., Peng J., Xiao Y., Li W., Tan L., Xu X., Qian Z. (2018). Porous Au@Pt Nanoparticles: Therapeutic Platform for Tumor Chemo-Photothermal Co-therapy and Alleviating Doxorubicin-induced Oxidative Damage. ACS Appl. Mater. Interfaces.

[25] Rastinehad A.R., Anastos H., Wajswol E., Winoker J.S., Sfakianos J.P., Doppalapudi S.K., Carrick M.R., Knauer C.J., Taouli B., Lewis S.C. (2019). Gold nanoshell-localized photothermal ablation of prostate tumors in a clinical pilot device study. Proc. Natl. Acad. Sci. USA.

[26] Peng J., Xiao Y., Li W., Yang Q., Tan L., Jia Y., Qu Y., Qian Z. (2018). Combined photothermal therapy and immunotherapy: Photosensitizer micelles together with IDO inhibitor enhance cancer photothermal therapy and immunotherapy. Adv. Sci..

[27] Zhao H., Xu J., Huang W., Zhan G., Zhao Y., Chen H., Yang X. (2019). Spatiotemporally light-activatable platinum nanocomplexes for selective and cooperative cancer therapy. ACS Nano.

[28] Huang X., Yin Y., Wu M., Zan W., Yang Q. (2019). LyP-1 peptide-functionalized gold nanoprisms for SERRS imaging and tumor growth suppressing by PTT induced-hyperthermia. Chin. Chem. Lett..

[29] Wu H., Wang C., Sun J., Sun L., Wan J., Wang S., Gu D., Yu C., Yang C., He J. (2019). Self-assembled and self-monitored sorafenib/indocyanine green nanodrug with synergistic antitumor activity mediated by hyperthermia and reactive oxygen species-induced apoptosis. ACS Appl. Mater. Interfaces.

[30] Zhao J., Chen J., Ma S., Liu Q., Huang L., Chen X., Lou K., Wang W. (2018). Recent developments in multimodality fluorescence imaging probes. Acta Pharm. Sin. B.

[31] Chen Z., Zhao P., Luo Z., Zheng M., Tian H., Gong P., Gao G., Pan H., Liu L., Ma A. (2016). Cancer cell membrane-biomimetic nanoparticles for homologous-targeting dual-modal imaging and photothermal therapy. ACS Nano.

[32] Zheng X., Xing D., Zhou F., Wu B., Chen W.R. (2011). Indocyanine green-containing nanostructure as near infrared dual-functional targeting probes for optical imaging and photothermal therapy. Mol. Pharm..

[33] Porcu E.P., Salis A., Gavini E., Rassu G., Maestri M., Giunchedi P. (2016). Indocyanine green delivery systems for tumour detection and treatments. Biotechnol. Adv..

[34] Nakamura M., Hayashi K., Nakamura J., Mochizuki C., Murakami T., Miki H., Ozaki S., Abe M. (2020). Near-infrared fluorescent thiol-organosilica nanoparticles that are functional-ized with IR-820 and their applications for long-term imaging of in situ labeled cells and depth-dependent tumor in vivo imaging. Chem. Mater..

[35] Fan W., Yung B., Huang P., Chen X. (2017). Nanotechnology for multimodal synergistic cancer therapy. Chem. Rev..

[36] Li C., Wang J., Wang Y., Gao H., Wei G., Huang Y., Yu H., Gan Y., Wang Y., Mei L. (2019). Recent progress in drug delivery. Acta Pharm. Sin. B.

[37] Wang D., Li X., Li X., Kang A., Sun L., Sun M., Yang F., Xu C. (2019). Magnetic and pH dual-responsive nanoparticles for synergistic drug-resistant breast cancer chemo/photodynamic therapy. Int. J. Nanomed..

[38] De Vita A., Liverani C., Molinaro R., Martinez J.O., Hartman K.A., Spadazzi C., Miserocchi G., Taraballi F., Evangelopoulos M., Pieri F. (2021). Lysyl oxidase engineered lipid nanovesicles for the treatment of triple negative breast cancer. Sci. Rep..

[39] Alvi S.B., Rajalakshmi P.S., Jogdand A.B., Nazia B., Bantal V., Rengan A.K. (2022). Chitosan IR806 dye-based polyelectrolyte complex nanoparticles with mitoxantrone combination for effective chemo-photothermal therapy of metastatic triple-negative breast cancer. Int. J. Biol. Macromol..

[40] Yin Y., Ben Hu B., Yuan X., Cai L., Gao H., Yang Q. (2020). Nanogel: A Versatile nano-delivery system for biomedical applications. Pharmaceutics.

[41] Yang Q., Peng J., Xiao H., Xu X., Qian Z. (2022). Polysaccharide hydrogels: Functionalization, construction and served as scaffold for tissue engineering. Carbohydr. Polym..

[42] Fang J., Islam W., Maeda H. (2020). Exploiting the dynamics of the EPR effect and strategies to improve the therapeutic effects of nanomedicines by using EPR effect enhancers. Adv. Drug Deliv. Rev..

[43] Yang Q., Xiao Y., Yin Y., Li G., Peng J. (2019). Correction to erythrocyte membrane-camouflaged IR780 and DTX coloading polymeric nanoparticles for imaging-guided cancer photo-chemo combination therapy. Mol. Pharm..

[44] Yang Q., Peng J., Kun Shi K., Xiao Y., Liu Q., Han R., Wei X., Qian Z. (2019). Rationally designed peptide-conjugated gold/platinum nanosystem with active tumor-targeting for enhancing tumor photothermal-immunotherapy. J. Control. Release.

[45] Peng J., Yang Q., Xiao Y., Shi K., Liu Q., Hao Y., Yang F., Han R., Qian Z. (2019). Tumor Microenvironment Responsive Drug-Dye-Peptide Nanoassembly for Enhanced Tumor-Targeting, Penetration, and Photo-Chemo-Immunotherapy. Adv. Funct. Mater..

[46] Zhang L., Tan L., Chen L., Chen X., Long C., Peng J., Qian Z. (2016). A simple method to improve the stability of docetaxel micelles. Sci. Rep..

[47] Hu S., Lee E., Wang C., Wang J., Zhou Z., Li Y., Li X., Tang J., Lee D.H., Liu X. (2015). Amphiphilic drugs as surfactants to fabricate excipient-free stable nanodispersions of hydrophobic drugs for cancer chemotherapy. J. Control. Release.

[48] Fan L., Zhang B., Xu A., Shen Z., Guo Y., Zhao R., Yao H., Shao J.W. (2018). Carrier-free, pure nanodrug formed by the self-assembly of an anticancer drug for cancer immune-therapy. Mol. Pharm..

[49] Li Y., Lin J., Cai Z., Wang P., Luo Q., Yao C., Zhang Y., Hou Z., Liu J., Liu X. (2020). Tumor microenvironment-activated self-recognizing nanodrug through directly tailored assembly of small-molecules for targeted synergistic chemotherapy. J. Control. Release.

[50] Gou S., Yang J., Ma Y., Zhang X., Zu M., Kang T., Liu S., Ke B., Xiao B. (2020). Multi-responsive nanococktails with programmable targeting capacity for imaging-guided mitochondrial phototherapy combined with chemotherapy. J. Control. Release.

[51] Zhao J., Zhang L., Qi Y., Liao K., Wang Z., Wen M., Zhou D. (2021). NIR laser responsive nanoparticles for ovarian cancer targeted combination therapy with dual-modal imaging guidance. Int. J. Nanomed..

[52] Su Y., Liu Y., Xu X., Zhou J., Xu L., Xu X., Wang D., Li M., Chen K., Wang W. (2018). On-demand versatile prodrug nanomicelle for tumor-specific bioimaging and photothermal-chemo synergistic cancer therapy. ACS Appl. Mater. Interfaces.

[53] Pham P.T.T., Le X.T., Kim H., Kim H.K., Lee E.S., Oh K.T., Choi H.G., Youn Y.S. (2020). Indocyanine green and curcumin co-loaded nano-fireball-like albumin nanoparticles based on near-infrared-induced hyperthermia for tumor ablation. Int. J. Nanomed..

[54] Feng B., Niu Z., Hou B., Zhou L., Li Y., Yu H. (2019). Enhancing triple negative breast cancer immunotherapy by ICG-templated self-assembly of paclitaxel nanoparticles. Adv. Funct. Mater..

[55] Liu K., Xing R., Zou Q., Ma G., Möhwald H., Yan X. (2016). Simple peptide-tuned self-assembly of photosensitizers towards anticancer photodynamic therapy. Angew. Chem. Int. Ed..

[56] Guo Y., Jiang K., Shen Z., Zheng G., Fan L., Zhao R., Shao J. (2017). A small molecule nanodrug by self-assembly of dual anticancer drugs and photosensitizer for synergistic near-infrared cancer theranostics. ACS Appl. Mater. Interfaces.

[57] Zhao R., Zheng G., Fan L., Shen Z., Jiang K., Guo Y., Shao J.W. (2018). Carrier-free nanodrug by co-assembly of chemotherapeutic agent and photosensitizer for cancer imaging and chemo-photo combination therapy. Acta Biomater..

[58] Li Y., Liu G., Ma J., Lin J., Lin H., Su G., Chen D., Ye S., Chen X., Zhu X. (2017). Chemotherapeutic drug-photothermal agent co-self-assembling nanoparticles for near-infrared fluorescence and photoacoustic dual-modal imaging-guided chemo-photothermal synergistic therapy. J. Control. Release.

[59] Lin J., Li C., Guo Y., Zou J., Wu P., Liao Y., Zhang B., Le J., Zhao R., Shao J.W. (2019). Carrier-free nanodrugs for in vivo NIR bioimaging and chemo-photothermal synergistic therapy. J. Mater. Chem. B.

[60] Zhang J., Zhao B., Chen S., Wang Y., Zhang Y., Wang Y., Wei D., Zhang L., Rong G., Weng Y. (2020). Near-infrared light irradiation induced mild hyperthermia enhances glutathione depletion and DNA interstrand cross-link formation for efficient chemotherapy. ACS Nano.

